# Efficient electroorganic synthesis of 2,3,6,7,10,11-hexahydroxytriphenylene derivatives

**DOI:** 10.3762/bjoc.8.196

**Published:** 2012-10-10

**Authors:** Carolin Regenbrecht, Siegfried R Waldvogel

**Affiliations:** 1 Institute for Organic Chemistry, Mainz University, Duesbergweg 10–14, 55128 Mainz, Germany

**Keywords:** catechol, electrochemical oxidation, hexahydroxytriphenylene, ketals, propylene carbonate

## Abstract

2,3,6,7,10,11-Hexahydroxytriphenylene of good quality and purity can be obtained via anodic treatment of catechol ketals and subsequent acidic hydrolysis. The electrolysis is conducted in propylene carbonate circumventing toxic and expensive acetonitrile. The protocol is simple to perform and superior to other chemical or electrochemical methods. The key of the method is based on the low solubility of the anodically trimerized product. The shift of potentials is supported by cyclic voltammetry studies.

## Introduction

The unique spectroscopic and geometric features of triphenylenes give rise to a variety of applications for this very common structural motif. The use of triphenylenes in discotic liquid crystals [1–2] as building blocks in supramolecular chemistry [3–4] as well as in solid-state chemistry [5] is well documented. Furthermore, triphenylenes are applied as components of functional polymers [6–7] and fluorescent labels [8–9]. Typically, the oxidative trimerization of catechol derivatives can be induced by metal salts in high oxidation states, for example by molybdenum pentachloride [4,10]. Electrochemical methods can be applied to realize the oxidative trimerization since the formation of metal waste is avoided [11–12]. Furthermore, no transition metal cations, which promote the cleavage of the ketal moiety [11], are involved.

The anodic oxidation of catechol derivatives was first demonstrated by Parker et al. [13]. Applying this methodology in a specific electrolysis cell, Simonet et al. established a trimerization protocol [14–16]. Usually, yields are in a moderate range (≤35%) [15] when the electrolysis is performed on platinum or graphite anodes in anhydrous and non-nucleophilic electrolytes [14]. Poor yields are caused by the low oxidation potential of the products and the preference for over-oxidation followed by decomposition of the generated radical cations [11,16].

Since we have a specific interest in a large-scale access to derivatives of 2,3,6,7,10,11-hexahydroxytriphenylene, we were prompted to develop a sustainable protocol providing the target compound by electroorganic methods. Precipitation during electrolysis avoided over-oxidation of the electron-rich triphenylene derivatives [12]. Yields up to 62% were obtained when tetrabutylammonium tetrafluoroborate (TBABF_4_) in acetonitrile (ACN) was used as electrolyte [11]. To our delight, we found that the electrolysis is accomplished under practical galvanostatic conditions using platinum sheets as electrode materials. Due to the large potential window and outstanding solubility for common conducting salts, ACN is among the standard solvents for electrochemical purposes [17]. However, the application of ACN in electrochemical processes is adversely affected by its severe toxicity and significant costs. Therefore, switching to an environmentally benign and inexpensive solvent is highly desired. This work demonstrates that propylene carbonate (PC) successfully replaces ACN by fine-tuning of the supporting electrolytes.

## Results and Discussion

### Anodic oxidation of catechol ketals

**Scheme 1:**
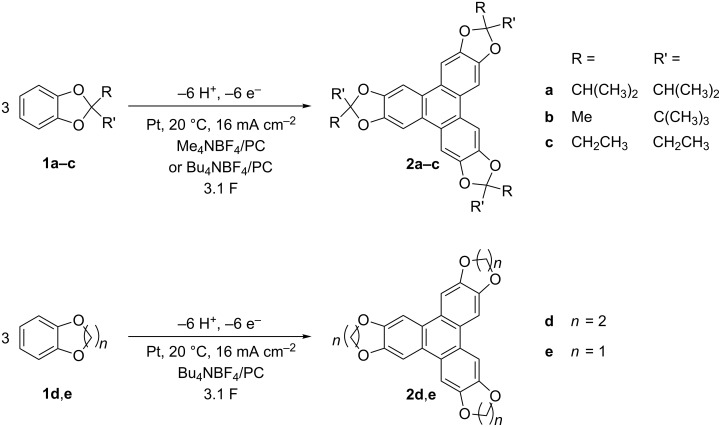
Experimental conditions for the anodic oxidation of catechol ketals.

We report a simple protocol for the anodic oxidation of catechol ketals forming triphenylene ketals (Scheme 1).

Best results were obtained in PC and TMABF_4_ (method **A**) or TBABF_4_ (method **B**) as electrolytes. Performing the reaction in PC is beneficial for several reasons: PC is hazard-free, inexpensive and suitable for large-scale reaction. Moreover, triphenylene ketals have lower oxidation potentials than the corresponding catechol ketals. Since the non-polar products exhibit a relative poor solubility in PC, precipitation is achieved during the electrolysis. Usually, the precipitate floats as fine particles in the cell and is prevented from over-oxidation. The best results for the individual catechol ketals are listed in Table 1.

**Table 1:** Anodic trimerization of catechol ketals.^a^

Entry	Catechol ketal	Method	Triphenylene ketal [%]		*CE* [%]

1	**1a** **1a**	A B	**2a** **2a**	80 70	52 45
2	**1b** **1b**	A B	**2b** **2b**	61 37	39 24
3	**1c** **1c**	A B	**2c** **2c**	61 50	39 32
4	**1d**	B	**2d**	29	25
5	**1e**	B	**2e**	39	19

^a^Conditions: Pt electrodes, *T* = 20 °C, *J* = 16 mA cm^−2^, 3.1 F/catechol ketal, argon.

Yields up to 80% are obtained for the dehydrotrimers **2a**–**c** in PC and TMABF_4_ as electrolyte. The solvation effects and polarity of the solvent greatly influence the precipitation of the desired products. Since the solvation increases with a decreasing size of the tetraalkylammonium cations, the polarity of the electrolyte increases as well [18–19]. The precipitation of the non-polar triphenylenes is promoted in the strongly polar electrolyte. Triphenylene derivatives **2d** and **2e** were obtained with 29 and 39% yield, respectively, using TBABF_4_ in PC. The yields are higher compared to that of reported electrochemical methods [15]. The simple reaction setup using an undivided cell and galvanostatic conditions applying this moderate current density allows a scale-up of this electroorganic synthesis.

### Cyclic voltammetry

**Figure 1:**
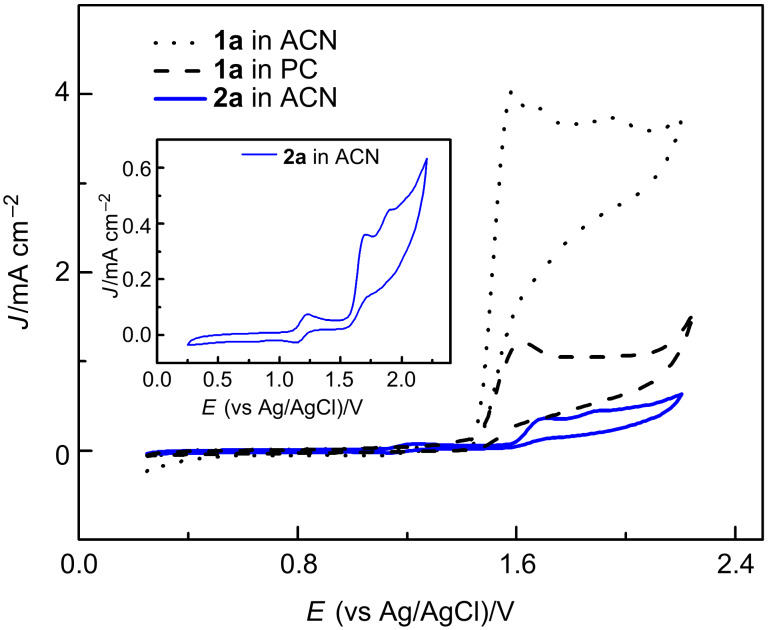
Cyclic voltammograms of catechol ketal **1a** in ACN and PC and triphenylene ketal **2a** in ACN, for magnification of **2a** see inner rectangle (**1a**: 5∙10^−3^ M in 0.1 M TBAClO_4_/ACN or 0.1 M TBAClO_4_/PC vs Ag/AgCl, **2a**: 5∙10^−3^ M in 0.1 M TBAClO_4_/ACN vs Ag/AgCl; sweep rates: 50 mV/s; third cycles).

Cyclic voltammetry studies show that triphenylene ketal **2a** gets more easily oxidized at lower potentials than subunit **1a** (Figure 1).

The first reversible process at *E*_ox_ = 1.22 V corresponds to the oxidation of **2a** to a radical cation [13]. This SET is followed by the irreversible oxidation at *E*_ox_ = 1.70 V. According to the Nernst equation, oxidation potentials should be increased due to the insolubility of **2a** in PC. Because of the insufficient solubility in this solvent, cyclic voltammograms were measured solely in ACN. The oxidation of **1a** was analyzed in PC and ACN. Both cyclic voltammograms show an irreversible oxidation at *E*_ox_ = 1.61 and 1.58 V, respectively [15]. The increase of the current densities beginning at 2.14 V is caused by the decomposition of electrolyte which was confirmed by blank experiments. The obtained data clearly indicate that triphenylene derivatives should precipitate during electrolysis to avoid over-oxidation.

### Preparation of 2,3,6,7,10,11-hexahydroxytriphenylene

**Scheme 2:**
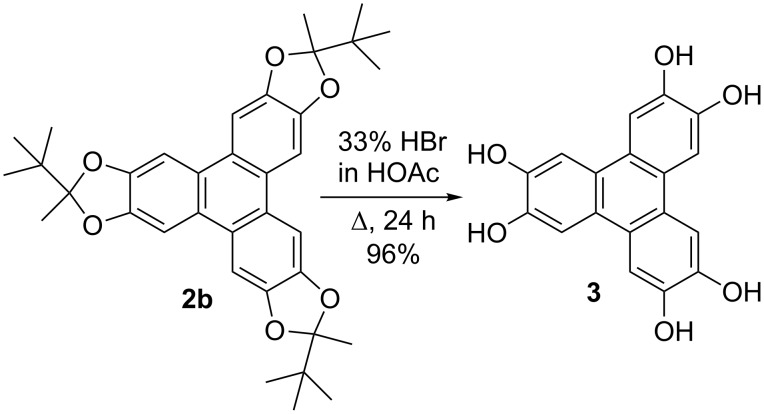
Acid-catalyzed cleavage of ketal moieties.

2,3,6,7,10,11-Hexahydroxytriphenylene (**3**) was obtained almost quantitatively by acidic cleavage of the ketal moieties of **2b** (Scheme 2).

The hydrolysis is initiated by the water which is present in the acetic acid. Commonly, **3** is prepared by dealkylation of 2,3,6,7,10,11-hexamethoxytriphenylene [20–21]. The resulting product is usually a black solid being colorized by chinoide byproducts [20,22] which are not present in our method. However, our method provides easy access to pure **3** which can easily be separated and purified by simple filtration.

## Conclusion

Triphenylene ketals are easily available by anodic oxidation using a simple galvanostatic protocol. Best results are obtained when the synthesized dehydrotrimers are almost insoluble in the electrolyte. Precipitation occurs during the electrolysis when employing tetraalkylammonium salts in PC. This effectively prevents the desired products from over-oxidation. Since PC is environmentally benign and inexpensive, the procedure significantly improved the access to the triphenylene derivatives. Moreover, acid-catalyzed cleavage of triphenylene ketals provides 2,3,6,7,10,11-hexahydroxytriphenylene almost quantitatively in very good quality.

## Experimental

### General protocol for anodic oxidation

The catechol ketal (10 mmol) was mixed with the electrolyte (method **A**: 2.012 g TMABF_4_ and 23.3 mL PC; method **B**: 4.116 g TBABF_4_ and 20.7 mL PC) in an undivided standard electrolysis cell and stirred under argon for 5 min. At 20 °C a galvanostatic electrolysis with a current density of 16 mA cm^−2^ was performed on platinum foil as electrodes (2.2 cm × 3.2 cm). The polarity of electrodes was reversed every 15 min. During the electrolysis, vigorous stirring was necessary and the formation of triphenylene products as light brown precipitates could be observed. After application of 3000 C (3.1 F) the electrolyte and precipitate were removed from the cell by dissolving in dichloromethane. After removal of the solvent under reduced pressure, the crude product and PC were dissolved in 40 mL of hot methanol. Water (17 mL) was slowly added to the hot solution and the mixture was allowed to cool to room temperature. Subsequently, the mixture was kept at 5 °C for several hours. The precipitate was filtered off and washed with methanol:water (7:3, 25 mL) and dried (45 °C, 8·10^−3^ mbar). Triphenylene derivatives **2a**–**e** were unequivocally identified.

## Supporting Information

File 1 Characterization data and spectra of synthesized compounds.

## References

[1] Barón M (2001). Pure Appl Chem.

[2] Boden N, Bushby R J, Cooke G, Lozman O R, Lu Z (2001). J Am Chem Soc.

[3] Schopohl M C, Siering C, Kataeva O, Waldvogel S R (2003). Angew Chem, Int Ed.

[4] Waldvogel S R, Fröhlich R, Schalley C A (2000). Angew Chem, Int Ed.

[5] Côté A P, Benin A I, Ockwig N W, O'Keeffe M, Matzger A J, Yaghi O M (2005). Science.

[6] Ringsdorf H, Schlarb B, Venzmer J (1988). Angew Chem, Int Ed Engl.

[7] Rose A, Zhu Z, Madigan C F, Swager T M, Bulović V (2005). Nature.

[8] Siering C, Kerschbaumer H, Nieger M, Waldvogel S R (2006). Org Lett.

[9] Berlman I B (1971). Handbook of Fluorescence Spectra of Aromatic Molecules.

[10] Waldvogel S R, Wartini A R, Rasmussen P H, Rebek J (1999). Tetrahedron Lett.

[11] Waldvogel S R, Mirk D (2000). Tetrahedron Lett.

[12] Waldvogel S R, Mirk D, Herbrüggen J (2001). GDCh-Monographie.

[13] Berchgaard K, Parker V D (1974). J Am Chem Soc.

[14] Le Berre V, Carlier R, Tallec A, Simonet J (1983). J Electroanal Chem.

[15] Chapuzet J-M, Simonet J (1991). Tetrahedron.

[16] Chapuzet J-M, Simonet-Guégen N, Taillepied I, Simonet J (1991). Tetrahedron Lett.

[17] Beck F (1974). Elektroorganische Chemie.

[18] Fry A J (2005). Electrochem Commun.

[19] Fry A J (2006). Tetrahedron.

[20] Naarmann H, Hanack M, Mattmer R (1994). Synthesis.

[21] Krebs F C, Schiødt N C, Batsberg W, Bechgaard K (1997). Synthesis.

[22] Voisin E, Williams V E (2008). Macromolecules.

