# Supplementary material for: Efficient electroorganic synthesis of 2,3,6,7,10,11-hexahydroxytriphenylene derivatives
Source: Beilstein J Org Chem. 2012 Oct 10;8:1721–4. doi: 10.3762/bjoc.8.196 (PMC3511005; doi:10.3762/bjoc.8.196)
Supplement: File 1 — Characterization data and spectra of synthesized compounds. [file Beilstein_J_Org_Chem-08-1721-s001.pdf]

Supporting Information

for

Efficient electroorganic synthesis of 2,3,6,7,10,11-hexahydroxytriphenylene derivatives

Carolin Regenbrecht and Siegfried R. Waldvogel*

Address: Institute for Organic Chemistry, Mainz University, Duesbergweg 10–14, 55128 Mainz, Germany

Email: Siegfried R. Waldvogel* - waldvogel@uni-mainz.de

* Corresponding author

Characterization data and spectra of synthesized compounds

A. Materials and methods S2

B. Experimental procedures and characterization S3

C. ^{1}H and ^{13}C NMR spectra S6

D. References S11

A. Materials and methods

Catechol ketals **1a**–**c** were prepared according to a common protocol, using a Dean–Stark trap [1]. The obtained crude products were purified by distillation in vacuo. Compounds **1d**–**e** were purchased and used without further purification. For CV measurements PC and ACN were desiccated by molecular sieves. Tetrabutylammonium perchlorate ($TBAClO_4$/Fluka) and tetramethylammonium tetrafluoroborate ($TMABF_4$/Acros) were used as purchased. $TBABF_4$ was synthesized from $NaBF_4$ and Bu_4NHSO_3 [2].

Standard cyclic voltammetry was carried out in a conventional three electrode cell using a µ-Autolab Type III potentiostat (Metrohm AG, Herisau, Switzerland). A glassy carbon disc served as working electrode and a glassy carbon rod as counter electrode. As reference an Ag/AgCl electrode (silver wire in saturated LiCl/ethanol solution) was employed.

Melting points were determined with a Melting Point Apparatus B-545 (Büchi, Flawil, Switzerland) and were uncorrected. Microanalysis was performed with a Vario MICRO cube (Elementar-Analysensysteme Hanau, Germany). ^{1}H NMR and ^{13}C NMR spectra were recorded at 25 °C by using Bruker AC 300, DPX 300 or DPX 400 instruments (Analytische Messtechnik, Karlsruhe, Germany). Chemical shifts (δ) are reported in parts per million (ppm) relative to TMS as internal standard or traces of $CHCl_3$ in the corresponding deuterated solvent. Mass spectra were obtained by using a MAT8200, MAT95XL (Finnigan, Bremen, Germany) or MS50 (Kratos, Manchester, England) apparatus employing EI and by using a Quattro LC (Waters-Micromass) or Micro TOF (Bruker) apparatus employing HRMS (positive mode). IR data were obtained by using Alpha Fouriertransform spectrometer (Bruker Optik GmbH, Ettlingen, Germany.

B. Experimental procedures and characterization

2,2,7,7,12,12-Hexa(1-methylethyl)-triphenyleno[2,3-*d*;6,7-*d*';10,11-*d*'']-tris[1,3]dioxole (2a)

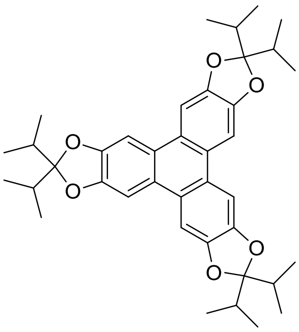

Yield: 1.636 g (2.67 mmol, 80%) of a pale brown solid. Mp >280 °C; ^{1}H NMR (400 MHz, $CDCl_3$) δ (ppm) 1.03 (d, ^3J = 6.8 Hz, 36H), 2.37 (sept, ^3J = 6.8 Hz, 6H), 7.64 (s, 6H); ^{13}C NMR (100 MHz, $CDCl_3$) δ (ppm) 16.2, 34.5, 99.6, 124.4, 124.8, 149.6; MS (EI, 70 eV) *m*/*z* (%): 612 (100) $[M]^{+\cdot}$, 569 (37), 263 (22); HRMS (EI, 70 eV) for $C_{39}H_{48}O_6$ calc.: 612.3451, found: 612.3451; elem. anal. $C_{39}H_{48}O_6 \cdot 0.5\ H_2O$ (621.79): calc. C 75.33 H 7.94, found: C 75.03 H 7.83.

2,7,12-Tri(1,1-dimethylethyl)-2,7,12-trimethyltriphenyleno[2,3-*d*;6,7-*d*';10,11-*d*'']-tris[1,3]dioxole (2b)

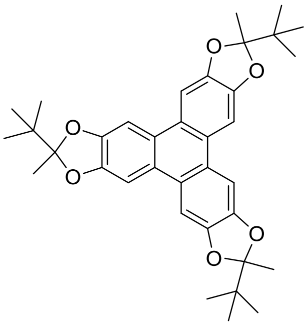

Yield: 1.152 g (2.02 mmol, 61%) of a pale brown solid. Mp: >280 °C; ^{1}H NMR (400 MHz, $CDCl_3$) δ (ppm) 1.12 (s, 27H), 1.65 (s, 9H), 7.66 (s, 6H); ^{13}C NMR (100 MHz, $CDCl_3$) δ (ppm) 20.5, 24.8, 39.8, 100.7, 124.0, 124.5, 148.4; MS (EI,

70 eV) *m*/*z* (%): 570 (100) $[M]^{+\cdot}$, 513 (85), 228 (38); HRMS (EI, 70 eV) for $C_{36}H_{42}O_6$ calc.: 570.2981, found: 570.2980; elem. anal. $C_{36}H_{42}O_6$ (570.72):calc. C 75.76 H 7.42, elem. anal. $C_{36}H_{42}O_6 \cdot 0.5$ MeOH (639.83): calc. C 74.72 H 7.56, found: C 74.62 H 7.28.

2,2,7,7,12,12-Hexaethyltriphenyleno[2,3-*d*;6,7-*d*';10,11-*d*'']tris[1,3]dioxole (2c)

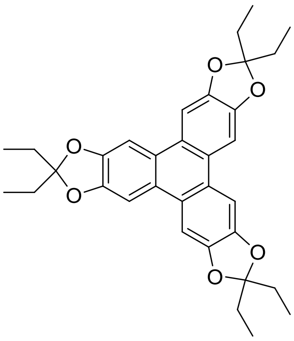

Yield: 1.074 g (2.03 mmol, 61%); Mp >280 °C; ^{1}H NMR (300 MHz, $CDCl_3$) δ (ppm) 1.03 (t, ^3J = 7.4 Hz, 18H), 2.01 (q, ^3J = 7.4 Hz, 12H), 7.67 (s, 6H); ^{13}C NMR (100 MHz, $CDCl_3$) δ (ppm) 7.3, 30.9, 100.6, 122.1, 124.5, 148.6; MS (EI, 70 eV) *m*/*z* (%): 528 (100) $[M]^{+\cdot}$, 499 (18), 235 (5); HRMS (EI, 70 eV) for $C_{33}H_{36}O_6$ calc.: 528.2512, found: 528.2501.

2,3,8,9,14,15-Hexahydrotriphenyleno[2,3-*d*;6,7-*d*';10,11-*d*'']tris[1,4]dioxine (2d)

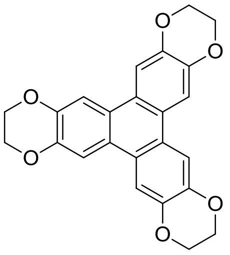

Yield: 0.386 g (0.96 mmol, 29%) of a pale brown solid. Mp >280 °C; ^{1}H NMR (400 MHz, DMSO-d_6) δ (ppm) 4.36 (s, 12H); 7.91 (s, 6H); ^{13}C NMR (100 MHz,

DMSO-d_6) δ (ppm) 64.3, 110.3, 123.2, 143.4; MS (EI, 70 eV) *m*/*z* (%): 402 (100) $[M]^{+\cdot}$, 346 (20), 201 (15); spectral data of **2d** match with [3].

Triphenyleno[2,3-*d*;6,7-*d*';10,11-*d*'']tris[1,3]dioxole (2e)

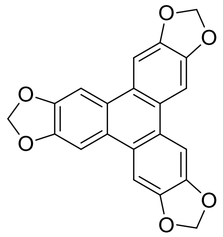

Yield: 0.466 g (1.29 mmol, 39%) of a brown solid. Totally insoluble, could not be identified by NMR [3]; MS (EI, 70 eV) *m*/*z* (%): 360 (100) $[M]^{+\cdot}$; mp >280 °C; IR (ATR): ν (cm^{-1}) = 1502, 1459 (m) ν(C=C_{aromat}), 1244, 1042 (s) ν(CO); 947 (s) δ(C_{aromat}-H), 851 (s) δ(C_{aromat}-H), 827 (s) δ(C_{aromat}-H); IR data match with [4].

Synthesis of 2,3,6,7,10,11-hexahydroxytriphenylene (3)

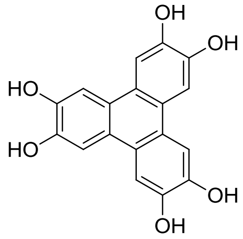

Compound **2b** (0.119 g, 0.21 mmol) was suspended in 33% HBr in acetic acid (15 mL) and heated to reflux for 24 h. The mixture was allowed to come to ambient temperature. The precipitate formed was filtered off, washed with water (10 mL) and vacuum-dried to yield a grey solid (0.065 g, 0.20 mmol, 96%). ^{1}H NMR (400 MHz, DMSO-d_6) δ (ppm) 7.61 (s, 6H), 9.28 (s, 6H); ^{13}C NMR (100 MHz, DMSO-d_6) δ (ppm) 107.8, 121.8, 145.2; MS (ESI, 10 eV): 347 $[M + Na]^+$, 325 $[M + H]^+$; IR (ATR): 3320 (br) ν(OH), 1442 (s) δ(OH), 1138 (s) ν(CO); spectral data match with [4].

C. ^{1}H and ^{13}C NMR spectra

Compound **2a**:

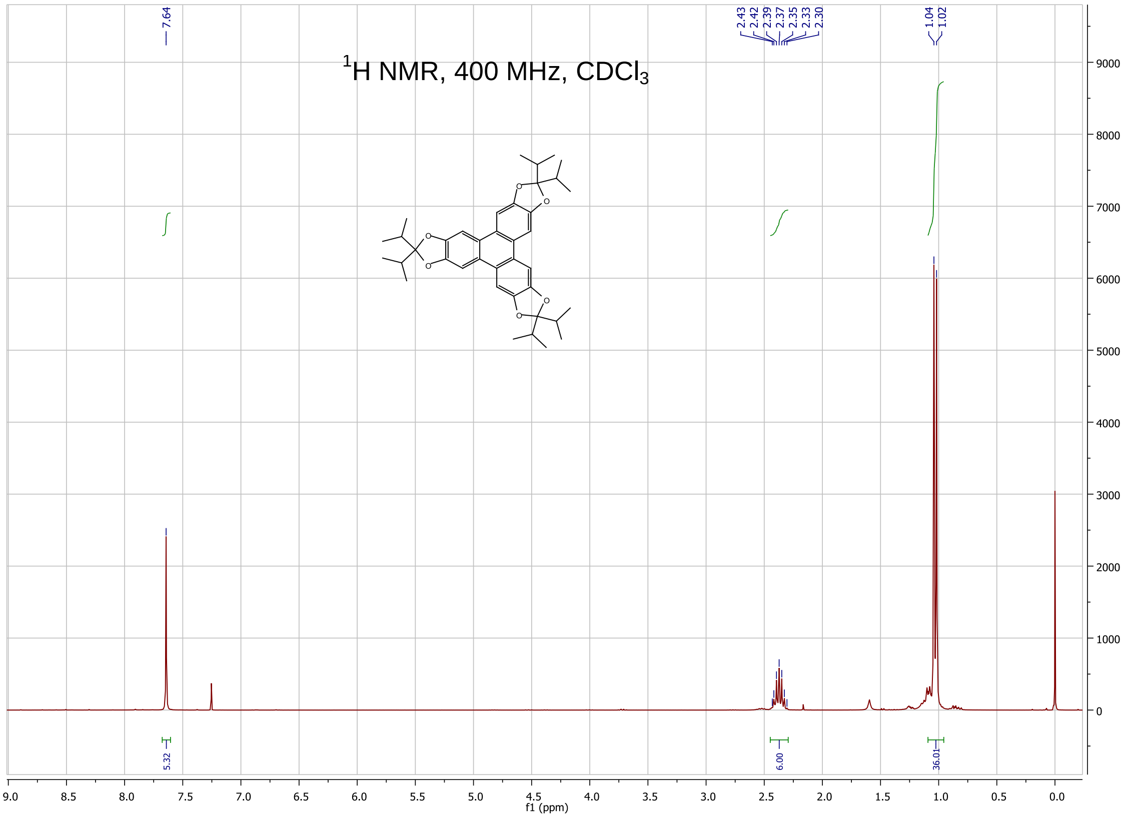

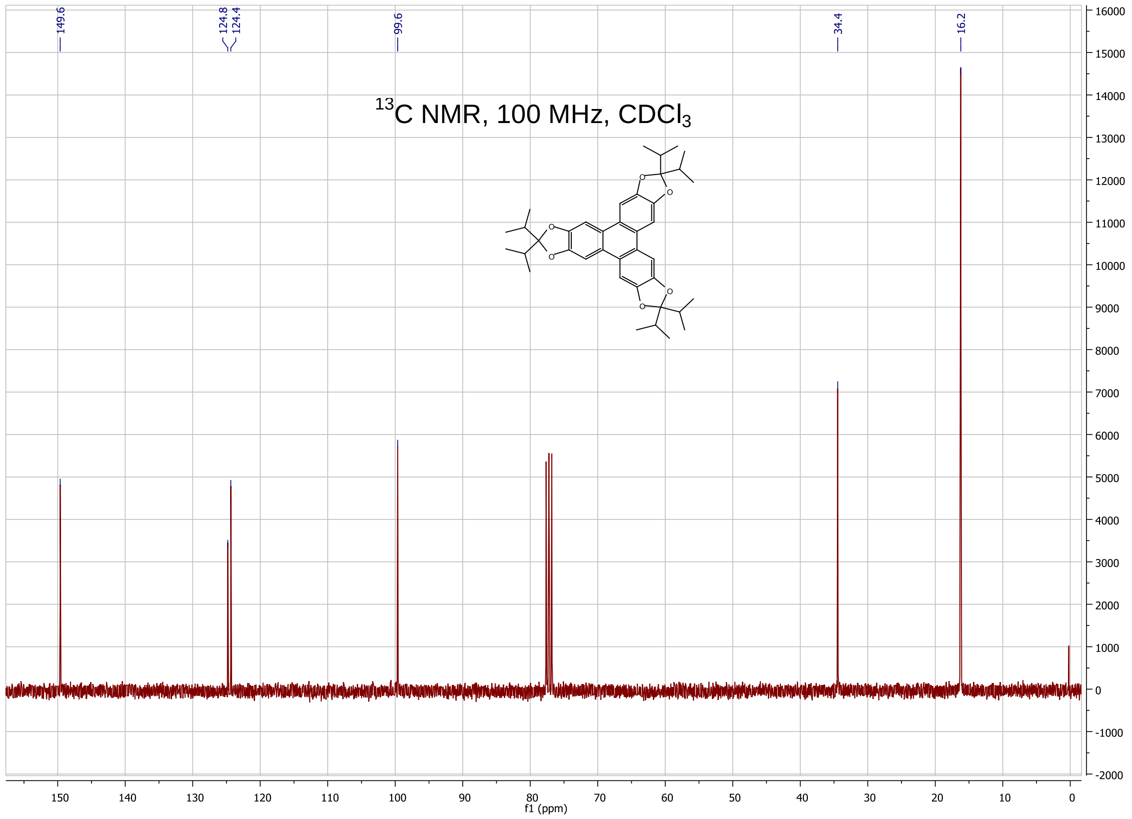

Compound **2b**:

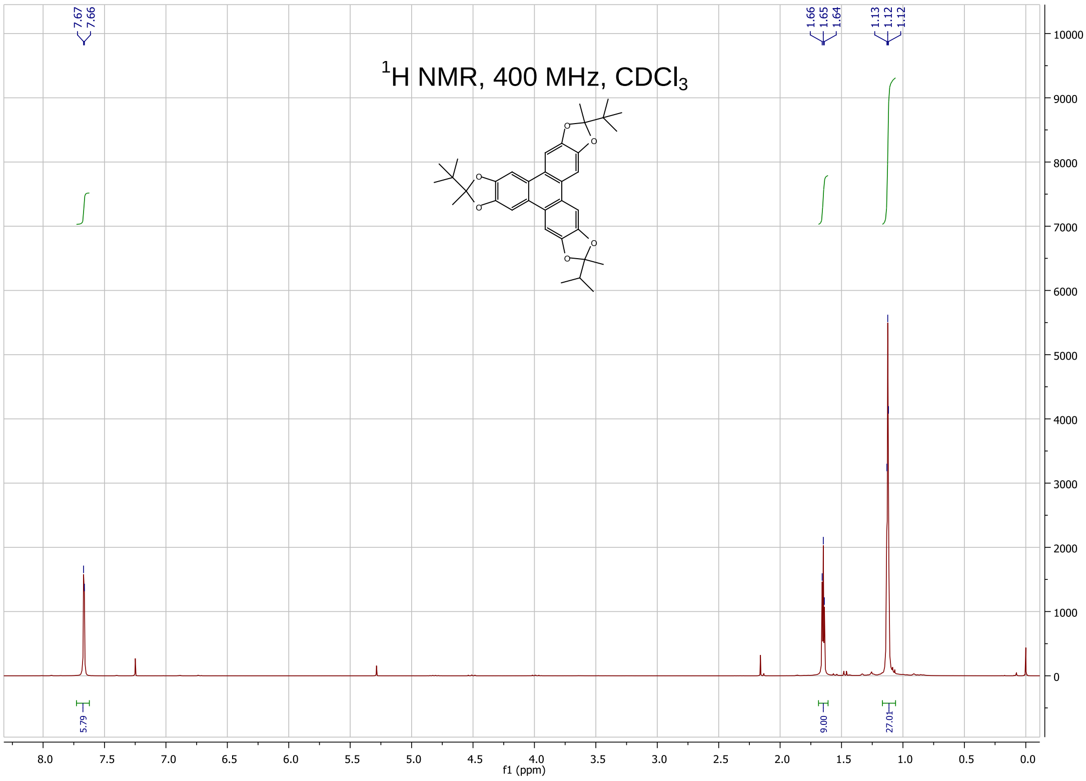

7.67
7.66
1.66
1.65
1.64
1.13
1.12
1.12
^{1}H NMR, 400 MHz, CDCl$_3$
5.79
9.00
27.01
f1 (ppm)

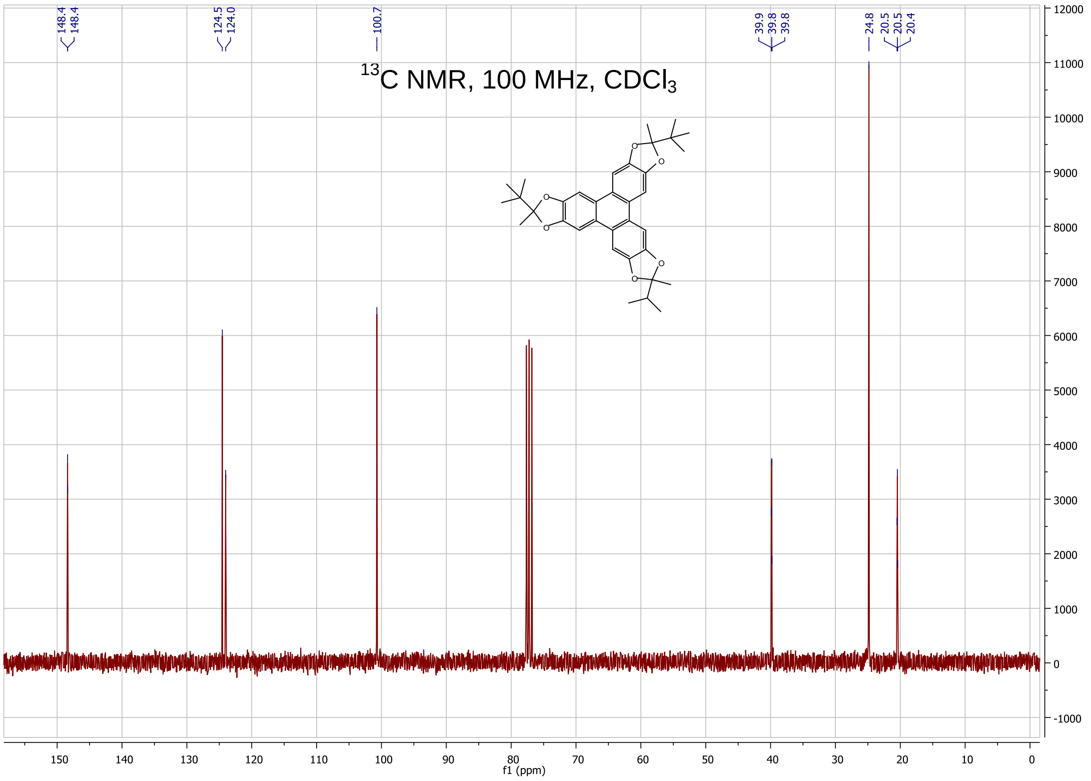

148.4
148.4
124.5
124.0
100.7
39.9
39.8
39.8
24.8
20.5
20.5
20.4
^{13}C NMR, 100 MHz, CDCl$_3$
f1 (ppm)

Compound **2c**:

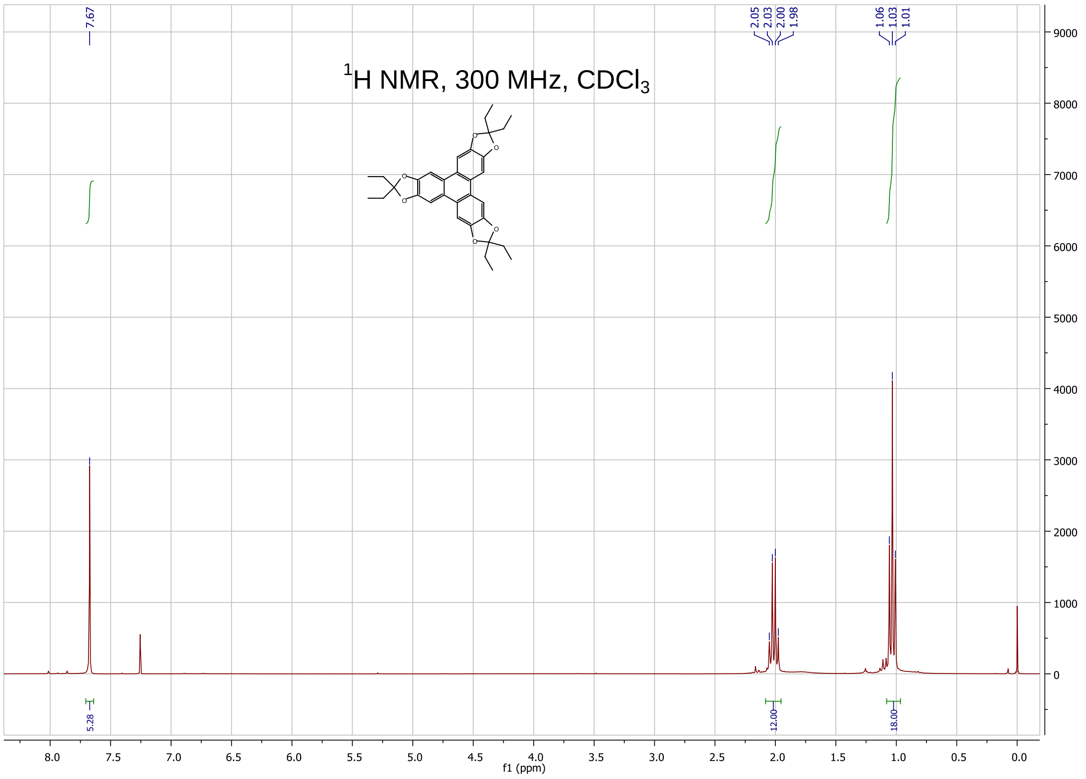

7.67
2.05
2.03
2.00
1.98
1.06
1.03
1.01
¹H NMR, 300 MHz, CDCl₃
5.28
12.00
18.00
f1 (ppm)

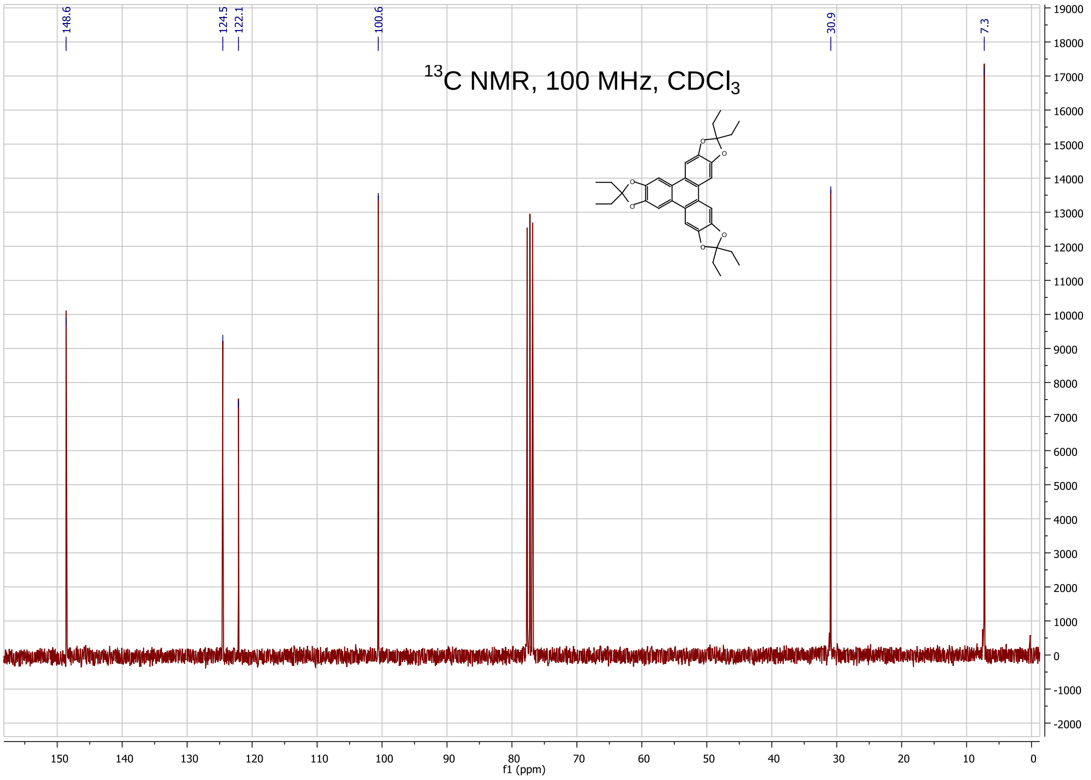

148.6
124.5
122.1
100.6
30.9
7.3
¹³C NMR, 100 MHz, CDCl₃
f1 (ppm)

Compound **2d**:

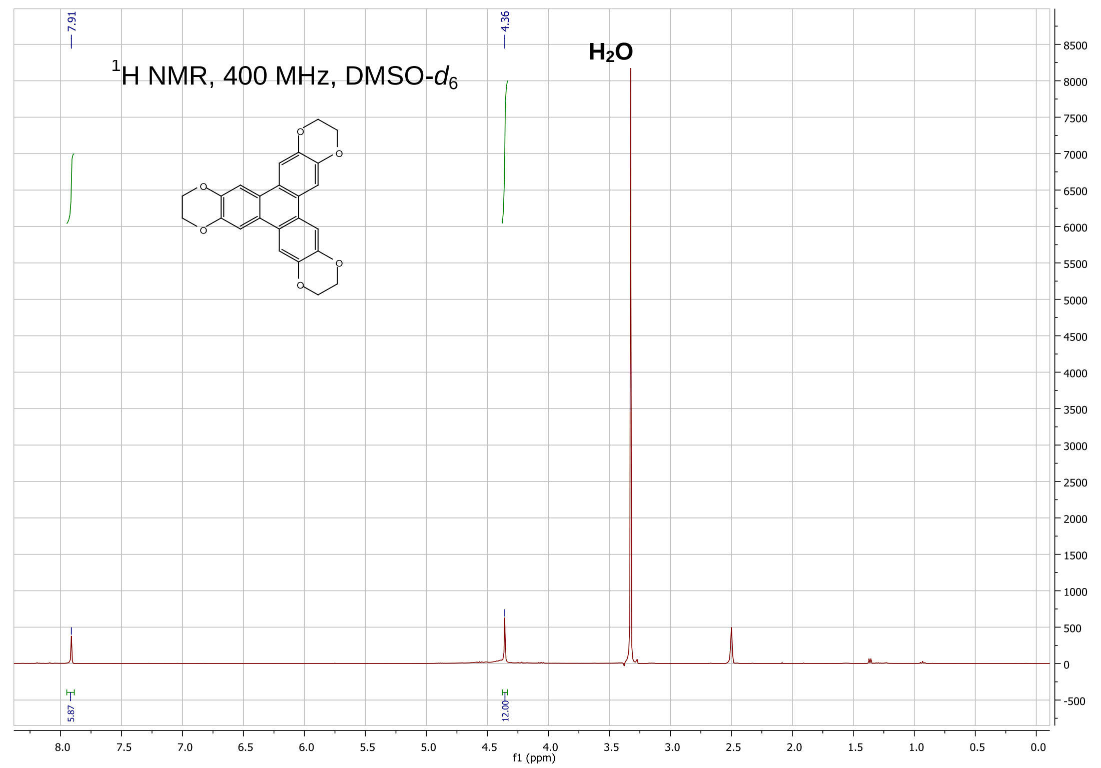

^{1}H NMR, 400 MHz, DMSO-d_6
H_2O
7.91
4.36
5.87
12.00
f1 (ppm)

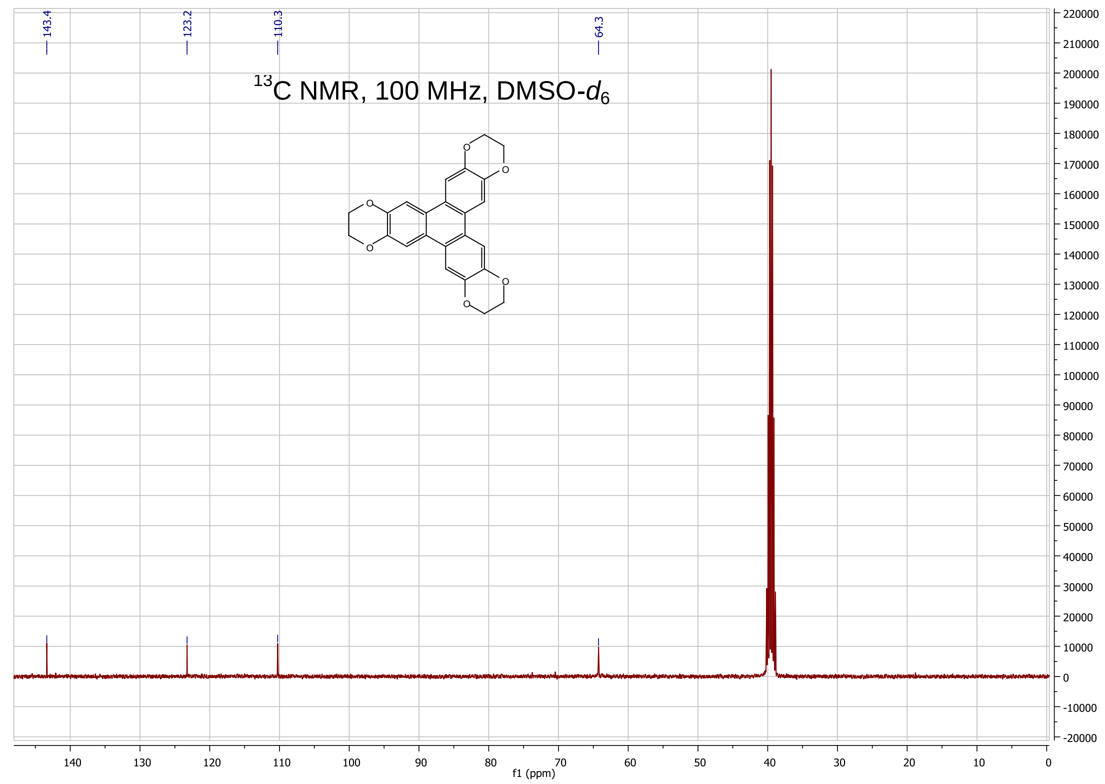

^{13}C NMR, 100 MHz, DMSO-d_6
143.4
123.2
110.3
64.3
f1 (ppm)

Compound **3**:

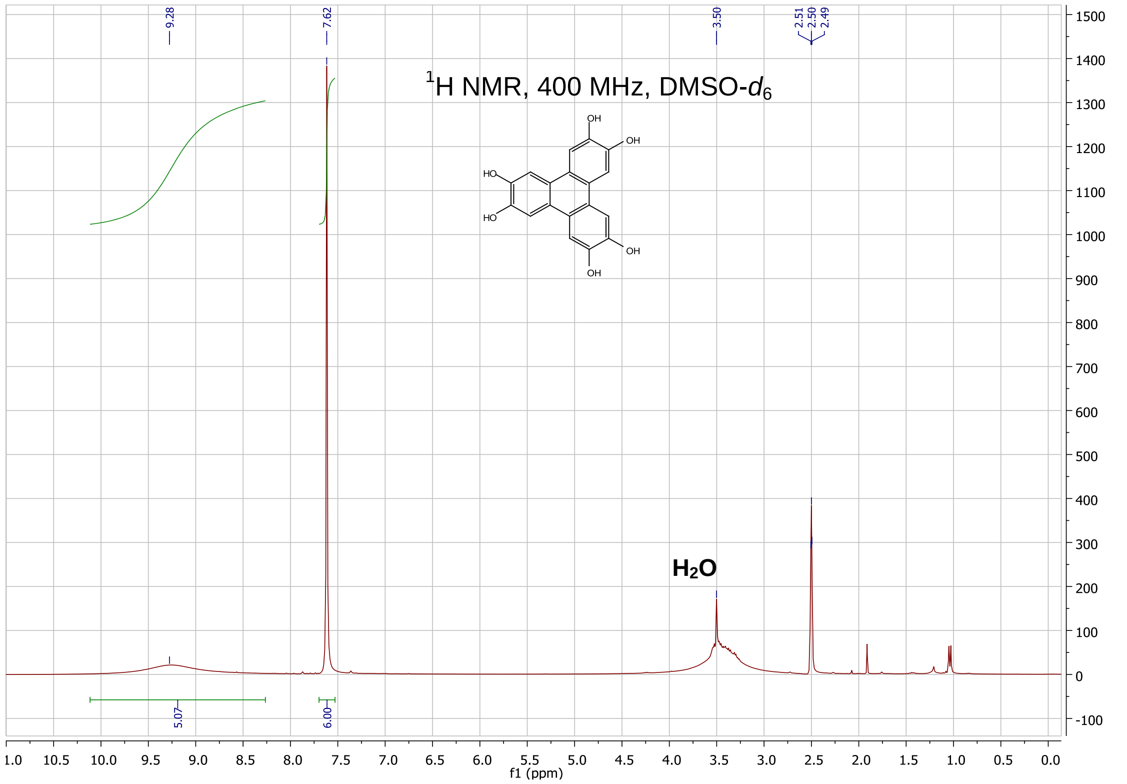
9.28
7.62
3.50
2.51
2.50
2.49
1H NMR, 400 MHz, DMSO-d6
OH
OH
HO
HO
OH
OH
H2O
5.07
6.00
f1 (ppm)

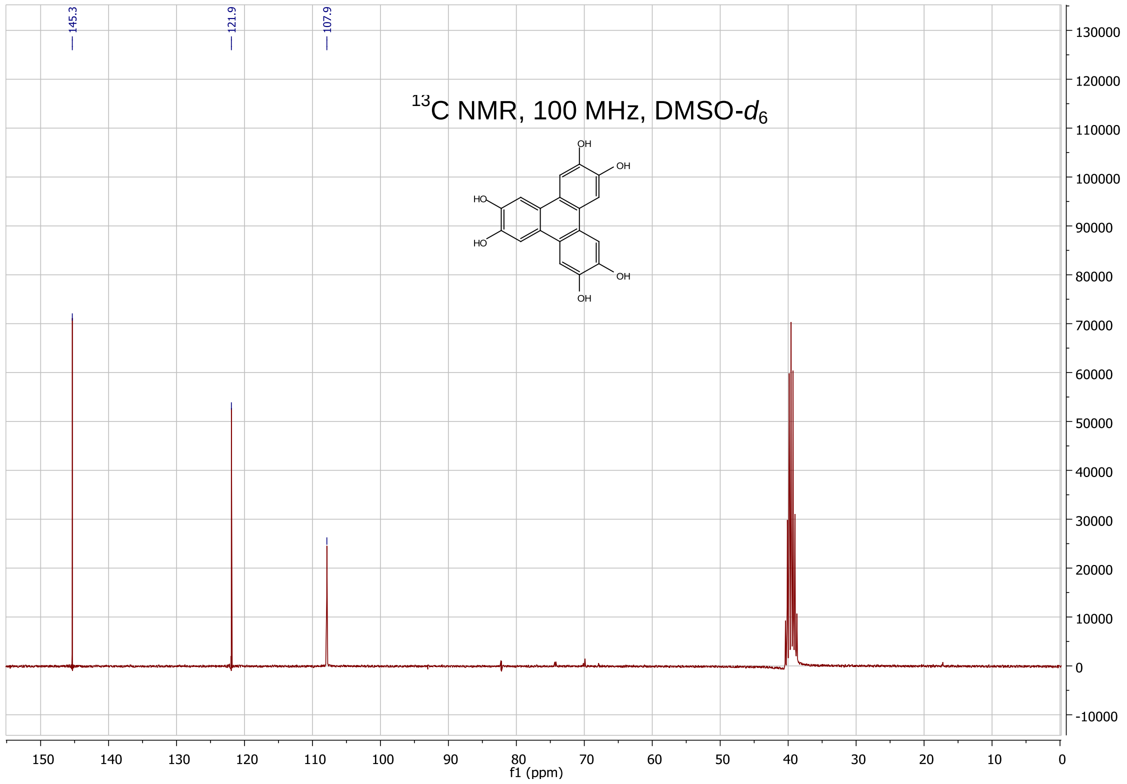
145.3
121.9
107.9
13C NMR, 100 MHz, DMSO-d6
OH
OH
HO
HO
OH
OH
f1 (ppm)

D. References

1. Boshta, N. M.; Bomkamp, M.; Waldvogel, S. R. *Tetrahedron* **2009,** *65,* 3773–3779. doi:10.1016/j.tet.2009.02.053
2. House, O.; Feng, E.; Peet, N. P. *J. Org. Chem.* **1971,** *36,* 2371–2375. doi:10.1021/jo00815a038
3. Chapuzet, J.-M.; Simonet, J. *Tetrahedron* **1991,** *47,* 791–798. doi:10.1016/S0040-4020(01)87068-X
4. Voisin, E.; Williams, V. E. *Macromolecules* **2008,** *41,* 2994–2997. doi:10.1021/ma800320j
